# Case of Traumatic Tetanus, Successfully Treated

**Published:** 1847-05

**Authors:** O. H. Costill

**Affiliations:** Frankfort, Pennsylvania


*Case of traumatic Tetanus, successfully treated.* By O. H. Costill, M. D. of Frankfort, Pennsylvania.

*Feb.* 18th, 1847. I was called about 10, A. M. to Margaret ——, a coloured woman, said to be in a fit. I found her lying on a settee, her arms, legs, and whole frame perfectly rigid. Her eyelids were drawn down so tightly as scarcely to be lifted, and her jaws were firmly set. Upon inquiry I found that two days previous she had received a carpet tack in the bottom of her right foot. It was withdrawn, and nothing more was thought of it till the evening previous, when she complained of pain in the foot and stiffness in the leg and back. She had, however, risen in the morning and resumed her usual occupation, until seized as above described. As the surface of her body was cold, I had her placed in bed and her clothes loosened, (though owing to the rigidity of her limbs they could not be removed,) and warm applications to the feet. After some time the spasm subsided. She opened her eyes, could move her limbs and converse. She complained of extreme tenderness in the anterior part of the sole of the foot; could scarcely bear it touched, though I could find no point where the nail had entered. The pain extended up the leg and back to the neck and jaws, and there was stiffness about the root of the tongue. I directed a lye poultice to the foot and a mixture of castor oil and turpentine to be given every hour and a half, until the bowels should be freely moved, intending after that to commence with opiates should the tetanic symptoms continue.

I saw her again at 12 o'clock. She was much more comfortable but had had one very severe spasm. Medicine had not operated. During the afternoon she became much worse. An urgent message was sent for me, and as I was absent from home my friend Dr. Taylor, at the request of my family, was on his way to visit her for me when I returned. He was so kind as to accompany me, and at my request attended the case with me subsequently.

It was about six in the evening when we saw her. She had had more than a dozen spasms during the afternoon, and one occurred while we were with her. Her breathing was difficult, as if choking, the jaws were set, foam proceeded from the mouth. The body was bent in a bow-shape resting upon the occiput and heels (opisthotonos.) Upon examining the foot after the spasm subsided, we could find no point where the nail had entered, but excessive tenderness on the anterior part of the sole within a space of the size of a dollar. Within this space I made two incisions crossing each other at right angles, into which lint saturated with spirits of turpentine was inserted, and the lye poultice re-applied. We directed her to take a pill containing two grains of opium and two of calomel every hour. We saw her again at half past 9 o'clock. She had taken two pills; had had no spasms—ordered one pill every two hours through the night, unless asleep. 19th, 9 A. M. Had taken four pills; had no spasms until six A. M., then had two, but not so severe; we applied at this time a solution of caustic potash along the spine. She was sick at the stomach this morning, and vomited light green fluid. To continue the pills, and as the bowels had not been moved since the attack, we directed a laxative enema. Evening. No spasms through the day; still sick, and vomiting occasionally; some pain in the limb extending up the back; neck and jaws better. The bowels have been freely moved three times. Mouth sore from the calomel. To discontinue the pills, and take two grains of opium at 10 o'clock, with nourishment through the night. 20th, 9 A. M. Slept some during the night, but was much distressed by sickness—vomiting the same light green fluid. Much more prostrate. The pulse, which had been nearly natural, till this morning, was frequent and feeble. To take a mixture containing 5 grains carb. ammonia and twenty drops tinct. opii every two hours; blister to the epigastrium. Evening. Much better; pulse full and soft. Sickness nearly gone. The blister had drawn but slightly. 21st, 9 A. M. Has slept well. No sickness—says she feels very well. To take the ammonia, &c., occasionally—from this time she continued to recover, and is now at service. The wound from the incision made in the foot continued sore for several days; but we could not say it suppurated.

I offer this case for publication, not because there was anything new in the means used for its cure; there certainly was not, but rather as additional evidence that this dreadful malady, so often fatal, will sometimes yield to the ordinary method of treatment. A case occurred to me many years since, in which the tetanic symptoms were very decided, though not so strong as in the case just related, which recovered by the liberal use of opium and stimuli and the dilatation of the wound.

*March 24th*, 1847.

